# Widespread cortical thinning, excessive glutamate and impaired linguistic functioning in schizophrenia: A cluster analytic approach

**DOI:** 10.3389/fnhum.2022.954898

**Published:** 2022-08-05

**Authors:** Liangbing Liang, Angélica M. Silva, Peter Jeon, Sabrina D. Ford, Michael MacKinley, Jean Théberge, Lena Palaniyappan

**Affiliations:** ^1^Graduate Program in Neuroscience, Western University, London, ON, Canada; ^2^Robarts Research Institute, Western University, London, ON, Canada; ^3^Department of Medical Biophysics, Western University, London, ON, Canada; ^4^London Health Sciences Centre, Victoria Hospital, London, ON, Canada; ^5^Lawson Health Research Institute, London, ON, Canada; ^6^Department of Psychiatry, Western University, London, ON, Canada; ^7^Department of Psychiatry, Douglas Mental Health University Institute, McGill University, Montreal, QC, Canada

**Keywords:** thickness, spectroscopy, computational linguistics, first-episode psychosis, natural language processing, formal thought disorder

## Abstract

**Introduction:**

Symptoms of schizophrenia are closely related to aberrant language comprehension and production. Macroscopic brain changes seen in some patients with schizophrenia are suspected to relate to impaired language production, but this is yet to be reliably characterized. Since heterogeneity in language dysfunctions, as well as brain structure, is suspected in schizophrenia, we aimed to first seek patient subgroups with different neurobiological signatures and then quantify linguistic indices that capture the symptoms of “negative formal thought disorder” (i.e., fluency, cohesion, and complexity of language production).

**Methods:**

Atlas-based cortical thickness values (obtained with a 7T MRI scanner) of 66 patients with first-episode psychosis and 36 healthy controls were analyzed with hierarchical clustering algorithms to produce neuroanatomical subtypes. We then examined the generated subtypes and investigated the quantitative differences in MRS-based glutamate levels [in the dorsal anterior cingulate cortex (dACC)] as well as in three aspects of language production features: fluency, syntactic complexity, and lexical cohesion.

**Results:**

Two neuroanatomical subtypes among patients were observed, one with near-normal cortical thickness patterns while the other with widespread cortical thinning. Compared to the subgroup of patients with relatively normal cortical thickness patterns, the subgroup with widespread cortical thinning was older, with higher glutamate concentration in dACC and produced speech with reduced mean length of T-units (complexity) and lower repeats of content words (lexical cohesion), despite being equally fluent (number of words).

**Conclusion:**

We characterized a patient subgroup with thinner cortex in first-episode psychosis. This subgroup, identifiable through macroscopic changes, is also distinguishable in terms of neurochemistry (frontal glutamate) and language behavior (complexity and cohesion of speech). This study supports the hypothesis that glutamate-mediated cortical thinning may contribute to a phenotype that is detectable using the tools of computational linguistics in schizophrenia.

## Introduction

Schizophrenia is a disorder that affects how language is employed in everyday use during social interactions (Covington et al., 2005; Kuperberg, 2010; Wible, 2012). Based on the Diagnostic and Statistical Manual of Mental Disorders 5th edition (DSM-5) (American Psychiatric Association, 2013), all of the 5 symptom criteria for diagnosing schizophrenia involve speech and language in one form or another (American Psychiatric Association, 2013). For example, hallucinations are often voices that speak (Alderson-Day et al., 2021); negative symptoms are characterized by “alogia” or reduced speech fluency; thought disorder is expressed as deviations in speech; catatonic features often include mutism (lack of speech production) (Oyebode, 2021); delusions often include an element of misinterpretation of social conversations or deficits in the use of propositional language (Zimmerer et al., 2017). Despite this strong linguistic dependency of the construct of schizophrenia, not every patient diagnosed with this illness displays a detectable speech disturbance (Roche et al., 2015; Kircher et al., 2018; Oomen et al., 2022). It is important to identify patients who are most likely to be afflicted in the language domain, as speech disturbances directly affect the educational and occupational success (Palaniyappan et al., 2019), interpersonal (Tan et al., 2014) and social functioning (Marggraf et al., 2020) as well as endured stigma (Penn et al., 2000). Identification of this subgroup may assist in prognostication in schizophrenia, as well as making early and targeted interventions for a group that may have higher educational and vocational needs possible, before they manifest significant deficits in these domains.

The heterogeneity of linguistic deficits may stem from the presence of a subgroup of patients who do not display the expected language anomalies (Oomen et al., 2022). Alternatively, conventional measures of “formal thought disorder (FTD)” that seek to examine overt communication difficulties may miss the subtle aspects of this deficit, thus introducing an apparent heterogeneity (Mikesell and Bromley, 2016). We need sensitive and objective measures of language indices to study this issue in detail (see Elvevåg et al., 2010; Foltz et al., 2016; Holmlund et al., 2020 for more explanations). One of these tools is natural language processing (NLP) in computational linguistics (Ratana et al., 2019; Corcoran and Cecchi, 2020; Corcoran et al., 2020; Hitczenko et al., 2021). NLP tools use computer algorithms to understand and analyze written text or speech. NLP is a branch of artificial intelligence that uses real-world language as input, and processes it using linguistic rules or patterns identified through statistics, to allow machines to make sense of our language. Such NLP tools do not rely on a clinician’s inferential skill to assess the cognitive-linguistic health status (Voleti et al., 2020) of patients from early stages of psychosis (Delvecchio et al., 2019) and are able to predict psychosis onset in individuals at clinical high-risk (CHR) (Bedi et al., 2015). These approaches have broadly focused on syntactic (Thomas et al., 1990; Thomas, 1996; Covington et al., 2005; Delvecchio et al., 2019) and semantic indices (Corcoran et al., 2018; Bar et al., 2019; Alonso-Sánchez et al., 2022; Parola et al., 2022) as the affected domains in psychosis.

Prior studies that focused on quantitative analysis of language have established the following dysfunctions in patients with schizophrenia. First, patients display syntactic simplification [(Morice and Ingram, 1982, 1983; Fraser et al., 1986; Morice and McNicol, 1986; King et al., 1990; DeLisi, 2001; Bilgrami et al., 2022) i.e., they use simple constructions with minimal clause dependencies and also with a limited richness of content]. Secondly, patients show patterns of reduced cohesion (Crider, 1997), for example, lacking prior reference when invoking a description (Chaika and Lambe, 1989) or insufficient lexical repetitions (Gupta et al., 2018) needed to generate cohesion during a discursive discourse (Crossley et al., 2016). Reduced syntactic complexity and cohesion can lead to aberrant word graphs (Mota et al., 2012) and a reduction in number of words spoken (reduced fluency) (Allen et al., 1993; DeLisi, 2001; Morgan et al., 2021).

While some of these features have been linked to the presence of clinically detected FTD, the rating-scale measures of FTD have been poor predictors of linguistic dysfunction *per se* (Mackinley et al., 2021; Tang et al., 2021). Furthermore, as symptom measures fluctuate over time (state-like), they have limited utility in identifying stable subgroups (Jablensky, 2006). Even among speech characteristics, those that relate to “positive symptoms” appear to be more state-related, while those relating to negative symptoms [or Impoverishment of Thinking (Liddle et al., 2002)] appear to be more pervasive. More trait-like measures, e.g., those derived from brain anatomy or genetic composition, that map on to emerging biological insights [e.g., implicating the glutamatergic synapses (Iyegbe and O’Reilly, 2022; Trubetskoy et al., 2022)], may be required to see if specific subgroups of patients have linguistic deficits. Furthermore, as antipsychotics themselves can induce language impairment (de Boer et al., 2020), recruitment of patients with first-episode psychosis with minimal exposure to antipsychotic medications is necessary to identify subgroups with language dysfunction from illness onset.

In the current study, we first identify subgroups of patients with first-episode schizophrenia using the neuroanatomical measure of MRI-derived cortical thickness. Structural neuroanatomical features are considered to be more stable than symptom rating and physiological recordings, which can vary on a day-to-day basis. In addition, MRI-derived thickness is quantified objectively in an automatized manner with minimal manual intervention in the quantification process. Thus, brain structure can provide more stable and reliable clustering solutions. Further, aberrant cortical thickness has been reported in various illness stages of schizophrenia (Zhao et al., 2022), and has been found to track the inter-individual differences in psychotic symptoms (Oertel-Knöchel et al., 2013) and Thought and Language Disorder scores in schizophrenia (Palaniyappan et al., 2020). Prior cluster analytic studies have uncovered a consistent cluster of patients with generalized reduction in cortical thickness (Dwyer et al., 2018; Chand et al., 2020; Liang et al., 2022). We use similar methods and then we examine if these subgroups have a meaningful neurochemical basis for their differences, by examining the MRS-derived glutamate levels measured from their frontal cortex, extending our recent work (Liang et al., 2022) to a larger sample.

Abnormal cortical thickness in schizophrenia has been previously linked to dysregulated glutamate levels (Plitman et al., 2014, 2016; Shah et al., 2020) and glutamatergic dysfunction had been considered to contribute to the “FTD” burden in schizophrenia (Kircher et al., 2018). We select dACC as our region of interest for glutamate measurement as it constitutes the core hub of the large-scale brain network called the Salience Network that appears to play a key role in the neurocognitive dysfunction in schizophrenia (Palaniyappan, 2021). Finally, we used a picture description task to study computational linguistic measures that are reflective of a “negative” FTD, first described by Fish (Casey and Kelly, 2019) and later reported by Andreasen (1979) and others (Kircher et al., 2018) as being more characteristic of established schizophrenia. Negative FTD is characterized by reduced quantity and quality of speech output; in a linguistically impoverished subgroup, this will be reflected in (i) reduced fluency (number of words spoken), (ii) reduced cohesion (measured by counting instances of content with prior reference, i.e., repeat content lemmas, e.g., run, running, and ran), and (iii) reduced syntactic complexity [mean length of sentences (MLS), clauses and minimal terminable units T-units, the smallest word group that could be considered a grammatical sentence, often composed of a main clause and subordinate clauses attached to it (Hunt, 1970)].

While there are numerous quantitative linguistic measures reported to be different in case-control comparisons, we chose items that predominantly map onto the negative symptom domain of schizophrenia (Tan et al., 2021; Bilgrami et al., 2022), independent of corpus-based distributional probabilities [which has limitations in understanding compositionality (Lenci, 2018)—a crucial locus of dysfunction in schizophrenia (Chaika, 1974)] and are readily interpretable [e.g., we did not use referential cohesion measure which is conflated in the presence of perseveration (Lundin et al., 2020)]. The features we selected are also intuitive in their link to known clinical features [reduced word count relates to alogia; lack of cohesion and simplified syntax relates to the poverty of content (Bedi et al., 2015; Corcoran et al., 2018; Minor et al., 2019)]. Furthermore, compared to other aspects of communication disturbances, the features of reduced fluency and richness of content (negative factor) selectively relate to poor response to treatment (Peralta et al., 1992). A neuroanatomically defined subgroup high in these “negative FTD type” linguistic features can be expected to be of prognostic relevance in schizophrenia.

Considering previous structural imaging-based cluster analytic studies, our primary hypothesis is that patient subgroups with distinct cortical thickness patterns can be identified in first-episode schizophrenia. In particular, a subgroup with widespread cortical thinning would emerge. Considering the association between cortical thinning, dysregulated glutamate levels and FTD burden, our secondary hypotheses are as follows: (i) The subgroup with deviant cortical thickness patterns also has abnormal glutamate levels measured in dACC; (ii) This subgroup displays impairments (negative FTD-type) in language production features, such as syntactic simplicity, reduced speech output and lower speech cohesion.

## Materials and methods

### Participants

We recruited 76 patients with first-episode psychosis from the Prevention and Early Intervention for Psychosis Program at the London Health Sciences Centre in London, Ontario, Canada from 2017 to 2021. Since 10 patients were unable to go through magnetic resonance imaging (MRI) scanning, we included data collected for 66 patients in this study. Inclusion criteria for patients include (1) having less than 14 days of lifetime exposure to antipsychotic medications, and (2) being at their first clinical presentation of psychotic symptoms. We followed up with patients for over 6 months to determine the validity of a diagnosis of first-episode schizophrenia prospectively. We also recruited 36 healthy volunteers, group-matched for age, sex, and parental socioeconomic status, who had no personal history of mental illnesses and no family history of psychotic disorders. All participants had no significant head injury, drug/alcohol dependence, or major medical illnesses, were fluent in English, and provided written informed consent to participate in the study. The work reported here is part of a longitudinal study registered on clinicaltrials.gov (Identifier: NCT02882204) and approved by the Western University Health Sciences Research Ethics Board, London, Ontario, Canada.

### Measures and instruments

#### Psychiatric symptoms

Symptom severity was measured by the 8-item Positive and Negative Syndrome Scale (PANSS) (Lin et al., 2018) through interviews conducted by two research psychiatrists. Functional outcome was indexed by the Social and Occupational Functional Assessment Scale (SOFAS) (Morosini et al., 2000). The duration of untreated psychosis was calculated using the first report of positive symptoms as the starting point. We also obtained patients’ NEET (Not in Education, Employment, and Training) status. We converted participants’ level of education into an ordinal scale (1: incomplete high school diploma; 2: completed high school diploma; 3: some post-secondary study; 4: completed post-secondary study or higher). Lifetime antipsychotic medication exposure was calculated by multiplying the number of days taking antipsychotics and prescribed Defined Daily Dose (DDD) values according to the World Health Organization (World Health Organization [WHO], 2022).

#### Thought and Language Index

Data was collected using Thought and Language Index (TLI) (Liddle et al., 2002) to reflect the two dimensions of language disorders in schizophrenia, impoverishment and disorganization. We used a picture-speech task that induced participants to elaborate 1-min spontaneous speech (oral soliloquies) in response to three images from the Thematic Apperception Test (Murray, 1943) after hearing specific instructions: “I am going to show you some pictures, one at a time. When I put each picture in front of you, I want you to describe the picture to me, as fully as you can. Tell me what you see in the picture, describe what you see in this image, and what you think might be happening.” Responses were recorded, transcribed, and scored. Impoverishment score was the sum of scores for these 3 dimensions: poverty of speech, weakening of goal and preservation of ideas, while disorganization score was indexed by 5 dimensions: looseness, peculiar use of words, peculiar sentences, peculiar logic, and distractibility.

#### Language assessment

The same transcribed speech samples also underwent automatic analysis to measure both syntactic complexity and cohesion at the semantic level.

##### Tool for the automatic analysis of syntactic complexity and sophistication

The automatic analysis of syntactic complexity and sophistication (TAASSC) is an open-source^1^ used in wide-ranging languages and grammatical frameworks with recent improvements in machine-learning approaches and NLP. This tool is complemented by a syntactic complexity analyzer (SCA)—a package with an accuracy of around 90% in part of speech (POS) tagging. The package includes a traditional and large measure of syntactic complexity following the taxonomy in Lu (2010): mean length of sentences (MLS), mean length of T-units (MLT), and mean length of clauses (MLC), word counts, and Terminal Units (T-unit) defined as the main clause with its attached subordinate clause(s) indicating speech cohesion as well as logical flow in the given information (see Supplementary material for more detailed descriptions).

##### Tool for the automatic analysis of cohesion

Tool for the automatic analysis of cohesion (TAACO 2.0)^2^ (Crossley et al., 2016) is a freely available text analysis tool which incorporates a wide-ranging of global indices—over 150 classic and recently developed indices related to text cohesion—local, global, and overall text cohesion can significantly predict both text cohesion and speaking quality whether the speaking samples show greater semantic overlap incorporating automated semantic analysis (Crossley et al., 2019). TAACO includes 194 indices of cohesion in seven main categories: Type token ratio (TTR) and density, lexical overlap (sentences), lexical overlap (paragraphs), semantic overlap, connectives, givenness, and source text similarity. Of this, we focus on the givenness index as we analyze speech rather than written text. Givenness, as opposed to newness in a discourse transcript, indicates whether information occurring in a segment has already occurred in an earlier segment. Repeat content words or lemmas (e.g., nouns, verbs, adjectives, etc.) are calculated as a proportion of the total number of words spoken within each 1-min picture description.

### Magnetic resonance imaging and magnetic resonance spectroscopy acquisition and processing

A total of 66 participants underwent neuroanatomy and spectroscopy scanning with an ultra-high-resolution 7-Tesla MRI scanner (8-channel transmit and 32-channel receive head-only coil) at Centre for Functional and Metabolic Mapping (CFMM), Western University, London, Canada. Structural images were obtained by a T1-weighted 0.75 mm isotropic MP2RAGE sequence with the following parameters: Repetition Time (TR) = 6,000 ms, Time to Echo (TE) = 2.83 ms, Inversion Time (TI)_1_ = 800 ms, TI_2_ = 2,700 ms, flip-angle 1 (α_1_) = 4°, flip-angle 2 (α_2_) = 5°, Field of View (FOV) = 350 mm × 263 mm × 350 mm, T_acq_ = 9 min 38 s, iPAT_PE_ = 3 and 6/8 partial k-space, slice thickness = 0.75 mm. Freesurfer (version 6.0.0) (FreeSurfer Software Suite, 2021) was used to preprocess the obtained T1-weighted images. FreeSurfer provides automated brain image processing steps including intensity normalization, tissue segmentation and cortical parcellation (recon-all Free Surfer Wiki, 2022). Visual inspections of errors such as surface location misplacement were carried out according to the troubleshooting guide provided by FreeSurfer team (FsTutorial/TroubleshootingData, 2022). We acquired the cortical thickness values based on the Destrieux parcellation atlas (Destrieux et al., 2010). Magnetic resonance spectroscopy (MRS) signal was measured on a voxel placed in the dorsal anterior cingulate cortex (dACC; MNI coordinates: 1, 16, 38). The details of MRS acquisition and analysis have been previously described (see Supplementary material) and a subset of this sample has been reported in prior works (Jeon et al., 2021; Liang et al., 2022).

### Statistical analyses

We applied agglomerative hierarchical clustering with Ward’s method and Euclidean distance to 148 cortical thickness values [based on Destrieux parcellation atlas (Destrieux et al., 2010) output using FreeSurfer] of all 102 participants including 66 patients and 36 healthy controls. Agglomerative hierarchical clustering starts with calculating the distance (e.g., Euclidean distance) between all pairs of data objects and putting the most similar data objects into the same cluster. The newly formed clusters are then again grouped with one another based on a linkage function (e.g., Ward’s method), until all data objects merge into one single cluster. The optimal number of clusters was determined by the consensus votes from 16 clustering validity indices using NbClust (Charrad et al., 2014) in R (version 4.0.3). Pearson’s chi-squared tests (with Yate’s continuity correction) were used to compare categorical variables, while Welch *t*-tests were used to compare continuous variables. If the obtained subgroups showed difference(s) in confounding variables (e.g., age or gender), ANCOVA was used to show effects between subgroups while accounting for effects of the covariates. We used FreeSurfer to find (1) between-cluster differences in vertex-by-vertex cortical thickness while regressing out the effect of age using a general linear model, and to locate (2) cortical regionals that correlated with glutamatergic metabolic levels. The thickness values at each vertex were mapped to the surface of an average brain template, and the cortical map was smoothed with a Gaussian kernel of 10 mm full width at half-maximum. We used Monte Carlo simulations with 1,000 permutations and a cluster-forming threshold of *P* = 0.05 (two-tailed) to correct for multiple comparisons as implemented in FreeSurfer.

## Results

Demographic, clinical, linguistic, and neurobiological measurements are provided in Table 1 and Supplementary material.

**TABLE 1:** Demographic, clinical, neurobiological, and linguistic data of patients with first-episode psychosis and healthy controls.

	FEP	HC	Pearson’s chi-squared test or Welch *t*-tests	
N	66	36	−	
**Demographics**				
Age (years)	22.82 (4.77)	21.53 (3.32)	*t*(94.043) = 1.6005, *p* = 0.1128	
Female/male	12/54	12/24	X-squared (1) = 2.1896, *p* = 0.1389	
Education scale (1/2/3/4)	15/18/20/13	5/3/14/13	X-squared (3) = 8.0131, *p* = 0.04574	*
**Clinical**				
PANSS-8 (total)	25.18 (6.72)	−	−	
PANSS-8 positive	11.62 (3.48)	−	−	
PANSS-8 negative	6.97 (4.41)	−	−	
PANSS-8 general	5.18 (2.46)			
DUP (weeks) [median (IQR)]	11.0 (4, 24)	−	−	
DDD lifetime exposure [median (IQR)]	0.5 (0, 2.99)	−	−	
Antipsychotic naïve (%)	42%			
**Functional**				
SOFAS	40.96 (12.40)	−		
NEET status: yes/no	24/29	0/31	X-squared (1) = 17.497, *p* < 0.0001	***
**Neurobiological**				
Glutamate (mM)	6.79 (1.16)	6.51 (1.35)	*t*(53.766) = 0.99493, *p* = 0.3242	
Mean cortical thickness (mm)	2.45 (0.12)	2.48 (0.096)	*t*(94) = 1.90350, *p* = 0.0600	
**Language variables**				
TLI (Total)	1.48 (1.41)	0.29 (0.39)	*t*(81.668) = 6.4188, *p* < 0.00001	***
TLI impoverishment	0.57 (0.72)	0.14 (0.25)	*t*(87.397) = 4.3669, *p* < 0.0001	***
TLI disorganization	0.91 (1.21)	0.15 (0.26)	*t*(75.114) = 4.9033, *p* < 0.00001	***
Average total number of words	119.18 (38.85)	141.34 (29.83)	*t*(88.706) = −3.1775, *p* = 0.002045	**
MLS	14.37 (4.58)	14.21 (2.74)	*t*(96.753) = 0.20899, *p* = 0.8349	
MLT	12.21 (3.00)	12.49 (2.08)	*t*(93.295) = −0.56025, *p* = 0.5767	
MLC	7.73 (1.20)	8.19 (1.18)	*t*(73.659) = −1.8858, *p* = 0.06327	
Repeated contents lemmas	0.229 (0.047)	0.247 (0.033)	*t*(89.792) = −2.1269, *p* = 0.03617	*

Values are reported as “Mean (SD)” unless specified otherwise.

IQR, Interquartile range; FEP, first episode psychosis; HC, healthy controls; PANSS, Positive and Negative Symptoms Scale; DUP, duration of untreated psychosis; DDD, Defined Daily Dose; SOFAS, Social and Occupational Functioning Assessment Scale; NEET, not in employment, education and training; TLI, Thought and Language Index; MLS, mean length of sentences; MLT, mean length of T-units; MLC, mean length of clauses.

*p < 0.05, **p < 0.01, ***p < 0.001.

The cluster validity procedure of hierarchical clustering of 148 cortical thickness values of 66 patients with first-episode psychosis and 36 healthy controls suggested that a two-cluster solution is optimal (9/16 cluster validity indices). Results of clustering only patients are shown in Supplementary material. Proceeding with a two-cluster solution, around 70% of patients (*n* = 46) with first-episode psychosis were clustered with the majority of the healthy controls (*n* = 33) in Cluster 1, while the remaining 30% of patients (*n* = 20) were in Cluster 2 which only included 3 healthy individuals. Demographic, clinical, neurometabolite, and language functioning information of the three subgroups (Cluster 1 patients, Cluster 2 patients, and Cluster 1 healthy controls) is summarized in Table 2 and Supplementary Table 1. Overall, compared to Cluster 1 patients, Cluster 2 patients have significantly older age, lower mean cortical thickens (non-significant age effect), higher glutamate concentration in dACC (non-significant age effect) as well as lower MLT (complexity) and repeated contents lemmas (cohesion) despite a preserved number of words within the given time frame (fluency). There is no significant difference between the two clusters in duration of untreated psychosis, lifetime exposure to antipsychotics, PANSS, and SOFAS scores.

**TABLE 2:** Demographic, clinical, neurobiological, and linguistic data of subgroups.

	Subgroup 1 patients	Subgroup 2 patients	Patient subgroup comparison	Subgroup 1 healthy controls
*N*	46	20		33
**Demographics**			**Pearson’s Chi-squared test or Welch *t*-tests**	
Age (years)	21.37 (3.72)	26.15 (5.31)	*t*(27.433) = −3.6527, *p* = 0.001081 *	21.15 (3.08)
Female/male	10/36	2/18	X-squared (1) = 0.62274, *p* = 0.43	12/21
Education scale (1/2/3/4)	9/14/16/7	6/4/4/6	X-squared (3) = 3.7761, *p* = 0.2867	5/3/14/10
**Clinical**			**Welch *t*-tests**	
PANSS-8 (total)	25.76 (7.02)	23.85 (5.91)	*t*(42.677) = 1.1376, *p* = 0.2616	−
PANSS-8 positive	11.67 (3.46)	11.50 (3.64)	*t*(34.519) = 0.18146, *p* = 0.8571	−
PANSS-8 negative	7.48 (4.46)	5.80 (4.15)	*t*(38.757) = 1.4755, *p* = 0.1481	−
PANSS-8 general	5.22 (2.41)	5.10 (2.63)	*t*(33.503) = 0.17063, *p* = 0.8655	
DUP (weeks) [median (IQR)]	13 (4, 26)	8.5 (5.75, 16.5)	*t*(23.362) = −0.53167, *p* = 0.6027	−
DDD lifetime exposure [median (IQR)]	0 (0, 2.54)	1.25 (0, 3.9)	*t*(20.156) = −1.6477, *p* = 0.1149	−
**Functional**			**Pearson’s Chi-squared test or Welch *t*-tests**	
SOFAS	40.98 (13.19)	40.90 (10.67)	*t*(44.354) = 0.025424, *p* = 0.9798	−
NEET status: yes/no	19/19	5/10	X-squared (1) = 0.62686, *p* = 0.4285	−
**Neurobiological**			**ANOVA with age as a covariate**	
Glutamate (mM)	6.57 (1.03)	7.28 (1.30)	*F*(1) = 5.10, *p* = 0.028, * Age effect: *p* = 0.13	6.50 (1.40)
Mean cortical thickness (mm)	2.50 (0.068)	2.32 (0.057)	*F*(1) = 126.225, *p* < 0.000, *** Age effect: *p* = 0.12	2.49 (0.061)
**Language variables**			**Welch *t*-tests**	
TLI (total)	1.28 (1.28)	1.93 (1.64)	*t*(29.517) = −1.5629, *p* = 0.1287	0.28 (0.40)
TLI impoverishment	0.48 (0.61)	0.79 (0.92)	*t*(26.725) = −1.3843, *p* = 0.1777	0.13 (0.23)
TLI disorganization	0.82 (1.14)	1.14 (1.37)	*t*(30.974) = −0.92366, *p* = 0.3628	0.16 (0.26)
Average total number of words	119.47 (35.45)	118.43 (47.46)	*t*(24.954) = 0.084721, *p* = 0.9332	141.53 (31.15)
MLS	14.58 (4.01)	13.91 (5.89)	*t*(23.59) = 0.4227, *p* = 0.6763	14.03 (2.67)
MLT	12.79 (3.09)	10.75 (2.20)	*t*(43.928) = 2.9509, *p* = 0.005066 **	12.45 (2.13)
MLC	7.90 (1.25)	7.30 (0.96)	*t*(40.658) = 2.0284, *p* = 0.04911 *	8.24 (1.21)
Repeated contents lemmas	0.240 (0.044)	0.204 (0.047)	*t*(28.741) = 2.6991, *p* = 0.01152 *	0.249 (0.034)

Values are reported as “Mean (SD)” unless specified otherwise.

IQR, Interquartile range; FEP, first episode psychosis; HC, healthy controls; PANSS, Positive and Negative Symptoms Scale; DUP, duration of untreated psychosis; DDD, Defined Daily Dose; SOFAS, Social and Occupational Functioning Assessment Scale; NEET, not in employment, education and training; TLI, Thought and Language Index; MLS, mean length of sentences; MLT, mean length of T-units; MLC, mean length of clauses.

*p < 0.05, **p < 0.01, ***p < 0.001.

**FIGURE 1:**
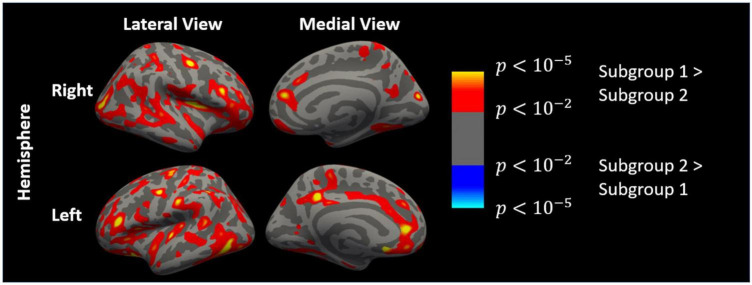
Cortical thickness map of differences between patients from Subgroup 1 and Subgroup 2 generated by FreeSurfer (regressing out age effect with a general linear model, uncorrected). Left hemisphere and right hemisphere in lateral and medial view, respectively.

Comparisons of cortical thickness between patients from the two subgroups (adjusted for age) are shown in Figure 1. After multiple testing corrections, patients in Cluster 1 had significantly lower thickness in 8 clusters (average area size = 410.44 mm^2^) in the left hemisphere and right hemisphere, respectively (see Supplementary Table 2 and Figure 1 for details). Comparisons of cortical thickness between the patients and controls from Cluster 1 (adjusted for age and corrected for multiple comparisons) revealed no regional differences in thickness values, indicating that this subgroup of patients had a “healthy” cortical morphological pattern.

**FIGURE 2:**
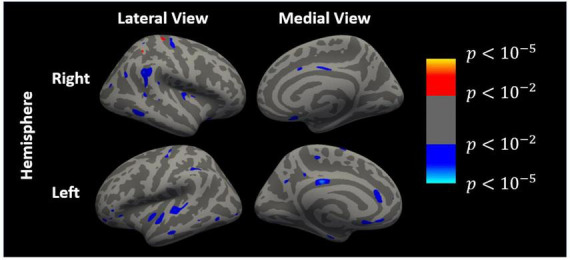
Cortical regions that are correlated with dACC glutamate levels (uncorrected) generated by FreeSurfer. Left and right hemispheres in lateral and medial view, respectively. Blue/cyan colors indicate negative correlations while red/yellow colors indicate positive correlations.

Multiple cortical regions were correlated with dACC glutamate levels in patients (Figure 2), but these correlations were not significant after multiple testing corrections. Correlation matrices of other variables of interest are presented in Supplementary material.

In summary, patients from Cluster 1 had similar neuroanatomical patterns to healthy controls, while patients from Cluster 2 were a distinct subgroup with widespread cortical thinning, higher glutamate concentration, and exhibited and reduced syntactic complexity and cohesion. This subgroup was thus impoverished in cortical structure as well as linguistic features.

## Discussion

In the current study, we identified a subgroup of 30% of patients with first-episode schizophrenia who are distinguishable on the basis of their MRI-derived cortical thickness profiles—displaying a generalized reduction in thickness (referred to as “Subgroup 2”) compared to the other group (70%) who have an unimpaired thickness profile similar to most healthy control subjects (referred to as “Subgroup 1”). Subgroup 2 is older in age at the time of the first presentation, has higher MRS-derived glutamate levels in the dorsal ACC and showed a pattern of linguistic impoverishment characterized by reduced fluency, syntactic simplicity, and repetitiveness. Taken together, these observations indicate a distinct subtype of schizophrenia that shows a pattern of cortical impoverishment along with linguistic impoverishment in the presence of higher prefrontal (dACC) glutamate levels at first presentation.

The emergence of a cortical impoverishment group showing a distributed reduction in cortical thickness compared to the other subgroup of patients and healthy controls is now a well-established feature of cluster analytical studies in schizophrenia. In a prior work where we studied two independent groups of patients with established schizophrenia as well as a part of the sample reported here, we observed a reliably identifiable subgroup of patients with cortical impoverishment (Liang et al., 2022), who did not differ from other patients in the cognitive or clinical severity. Similar findings also reported a “cortical impoverishment subgroup” at various illness stages (Sugihara et al., 2017; Dwyer et al., 2018; Chand et al., 2020; Pan et al., 2020), supporting the stability of this subtype. While the mechanistic processes underlying this structural deviation are still circumspect, based on the higher glutamate levels noted in this subgroup using 7T MRS from a dorsal ACC voxel, a putative link to excitotoxicity (Plitman et al., 2014) (or E/I dysfunction Limongi et al., 2020) can be drawn.

According to the NMDA hypofunction or glutamatergic dysregulation models of schizophrenia, higher glutamate transmission may relate to excitation-inhibition imbalance (Limongi et al., 2020) and if unchecked, may result in synaptic and neuronal loss (Wang and Qin, 2010). These cellular mechanisms have been hypothesized to underlie structural deficits in schizophrenia (Plitman et al., 2014). Multilevel genetic and physiological studies are needed to further pursue this observation. We now provide an important lead in this pursuit by identifying language dysfunction in this subgroup of schizophrenia. Additionally, we want to highlight the implications of dissecting neurobiological heterogeneity in schizophrenia. In our study, despite displaying similar symptom severity and social functioning, the two patient subgroups have distinct neurobiological underpinnings, and may represent different pathophysiological pathways of developing schizophrenia.

Through a parts-of-speech (POS) tagging approach in NLP, we studied “poverty of content” at 3 components of grammatical structures: mean length of sentences, clauses and T-units. All are large syntactic complexity indices used as a proxy of cognitive parameters because producing a T-unit is a more complex process than producing coordinated clauses (Szmrecsanyi, 2004). T-units serve as an informative index to distinguish the amount of independent clausal coordination in the expressed idea. Moreover, T-units provided the rule-based identification process considering the selecting word for subordination (e.g., using “because”) or coordination (e.g., using “and”) (Beaman, 1984). Therefore, a reduction in coordinated T-units demonstrates notable syntactic simplicity in our Subgroup 2. These results are congruent with Bilgrami and colleagues’ works (Bilgrami et al., 2022) who also reported lower POS syntactic complexity in those patients who had negative symptoms. The authors found that reduced sentence length and decreased use of words that introduce dependent clauses (e.g., using complementizer or determiner pronouns such as “that” and “which”) are associated with negative thought disorder (Bilgrami et al., 2022). Additionally, our observations raise the question of whether patients with higher developmental disruption form the subgroup with cortical and linguistic impoverishment since syntactic complexity is a phenomenon that develops during childhood (Givon, 2009; Frizelle et al., 2018) and reaches a plateau around the age of 20 (Nippold et al., 2014). If developmental disturbances during childhood and adolescence lie in the pathogenesis of schizophrenia and can be detected using NLP tools (*via* progressive aberrations in syntactic complexity; see Silva et al., 2022), this may provide a promising avenue for early identification.

In clinical settings, linguistic dysfunction in schizophrenia relies on a standardized rating scale (PANSS and TLI) to define speech impairment as one sign of FTD (Elvevåg et al., 2007; Iter et al., 2018). The two patient subgroups did not differ in TLI or PANSS scores even though the diagnostic group of FES differed from healthy subjects in TLI rating score as expected. This observation speaks to the ability of automated quantitative processes to parse the subtler aspects of language dysfunction, an issue that has been discussed at length in several recent works based on the NLP approach (Corcoran and Cecchi, 2020; Hitczenko et al., 2021). We observed a reduction of repeated content lemma (e.g., nouns, verbs, adjectives) in our Subgroup 2. This index traditionally characterizes the systematic relationship—explicit or implicit—between lexical items, i.e., cohesive cues, placed at the text surface (Sanders and Maat, 1976). For example, if two adjacent ideas (sentence-to-sentence, clause-to-clause) comprise the same noun (e.g., woman), the lexical repetition will explicitly help connect both ideas. However, if the first clause contains the word “bridge” and the second contains the word “iron,” the connection weakens even though it is logical. Therefore, in this work, we quantify cohesion (Halliday and Hasan, 1976; Graesser et al., 2004) through a lexical approach applied to how speech has been produced, without any assumption about how it is understood by listeners or readers (i.e., lexical cohesion as distinct from semantic coherence) (Just et al., 2020).

The linguistic phenomenon of reduced content word-lemmas relating to cortical thinning can be understood in several ways (Crossley et al., 2016). Firstly, reduced repetition of content-lemmas directly negatively influences the givenness of the generated speech. Givenness refers to the distribution of the given/known information or ideas as opposed to the new/unknown information. A “cortically impoverished” patient may build ideas as small clauses with little relationship between them. Secondly, a decline in the use of repeated content lemma makes it difficult to recover the meaningful information from the preceding passage, generating a sense of empty speech (i.e., poverty of content) with reduced informative value to the listener.

Our study has several strengths: We were able to overcome the difficulty of collecting speech data in an acute, untreated state of psychosis, and determine their diagnosis of first-episode schizophrenia. Furthermore, we ensured transcribers, as well as speech analysts, were blind to diagnosis. We employed ultra-high field strength MRS whereby the glutamate quantification from MS-spectra had a high specificity. Finally, we used multiple clustering procedures and derived a two-cluster solution based on a majority-based consensus, adding to the stability of the observed subtype. Nevertheless, several limitations need consideration. We had a limited number of female participants which limits generalizability; we did not see a statistical effect of sex between the groups, but our small numbers preclude a stratified analysis. Second, thickness-based clustering resulted in age differences between the subgroups; however, we included age as a covariate in downstream analyses for glutamate and regional thickness to ensure this confound did not affect the inferences we make. Nevertheless, the non-linear influence of age on these variables cannot be ruled out. Third, we did not assess IQ formally. In our recent study where we examined the influence of cognition on thickness-based clustering in greater detail, the effect of individual differences in cognitive performance in the thickness profile was minimal among patients (Liang et al., 2022). Thus, while we can be confident that the reported thickness reduction and language dysfunction in a subgroup is not due to low extreme distributions of IQ as a result, we cannot exclude that an undetermined proportion of variance in these variables could be explained by cognitive differences. Finally, our speech samples were restricted to one language (English) and were based on a single discursive discourse (picture description) and single modality (oral soliloquies-monolog) elicited in the context of a research interview. The effect of contextual differences, language as well as types and duration of elicitation task on our linguistic observations needs further examination.

In sum, we can link the putative excitotoxicity (glutamate excess) to reduced gray matter thickness (cortical impoverishment) and the objectively computed negative phenomenology of language (or linguistic impoverishment) in first-episode schizophrenia. This finding supports the presence of detectable neurobiological subtypes of schizophrenia. Connecting the cellular/synaptic processes (glutamate) with objectively quantified language behaviors through macroscopic brain changes (thickness) may facilitate more consistent brain-behaviors mapping in schizophrenia.

## Data availability statement

The authors agree to make data and codes supporting the results presented in their paper available upon reasonable request, within the stipulations laid by the ethics committee. Requests to access the datasets should be directed to the corresponding author.

## Ethics statement

The studies involving human participants were reviewed and approved by the Western University Health Sciences Research Ethics Board. The patients/participants provided their written informed consent to participate in this study.

## Author contributions

LP supervised and planned the study. SF and MM recruited patients and collected the data. LL and MM conducted MRI data analysis. JT designed spectroscopy protocol, and supervised PJ for MRS data acquisition and analysis. AMS analyzed the speech sample data. LL designed and conducted the statistical analyses. LL and AMS created the original draft under supervision of LP. All authors contributed to and approved the final manuscript.

## Footnotes

1
https://www.linguisticanalysistools.org/taassc.html

2
https://www.linguisticanalysistools.org/taaco.html
