# Supplementary material for: Widespread cortical thinning, excessive glutamate and impaired linguistic functioning in schizophrenia: A cluster analytic approach
Source: Front Hum Neurosci. 2022 Aug 5;16:954898. doi: 10.3389/fnhum.2022.954898 (PMC9390601; doi:10.3389/fnhum.2022.954898)
Supplement: Supplementary file 1 [file Data_Sheet_1.pdf]

Supplementary Material

1 Additional sociodemographic and clinical information

	FEP	HC	Total
**N**	66	36	102
**Ethnicity**			
Black or African Canadian	10	0	10
Latino	1	0	1
Middle Eastern	3	0	3
Mixed race	1	1	2
Native Canadian	1	0	1
South/East Asian	4	12	16
White	46	23	69
**Antipsychotic medication**			
Abilify	8		
Clozapine	1		
Invega	6		
Olanzapine	9		
Rexulti	2		
Risperidone	12		
None	28		

2 Language Metrics

Measures	Dimensions	Detailed Descriptions
**Thought and Language Index**		
Impoverishment	Poverty of speech	Speech productions lack details and elaboration
	Weakening of goal	Lack of ideas and meaningful information
	Preservation of ideas	Repetitive contents, even if given different stimuli
Disorganization	Looseness	Lack of logical flow or connection of ideas
	Peculiar use of words	Invented or rarely used words
	Peculiar sentences	Unusual sentence structures that impede speech comprehension
	Peculiar logic	Reaching conclusions without enough evidence
	Distractibility	Distracted by external stimulus
**Syntactic Complexity (Production)**	Mean length of sentences (MLS)	Average number of words per sentence.
	Mean length of T-units (MLT)	Average number of words per T-unit. T-unit is defined as the main clause with its attached subordinate clause(s).

	Mean length of clauses (MLC)	Average number of words per clause.
**Cohesion**	Repeated contents lemmas	Average number of content words that are repeated at least once divided by the total number of words in the text

2.1 Patient Speech Data Examples

Example output of syntactic complexity. Traditional indices from Tool for the automatic analysis of syntactic complexity and sophistication (TAASSC)

ID	Picture	Transcribe speech	MeanMLS	MeanMLT	MeanMLC
FEPxxx	2	Uh there is a black and white sun seen in all the building all the big painted building It is wood there is three windows there is a girl that is looking down from the balcony there is water down on the water there is another an abandoned building there is a guy in a canoe there is lots of workers maybe gathering up some fish and that is it that is all I can get uh it is black and white pencil sketched	7.417	7.639	6.078

Example output of Textual cohesion based upon the givenness index. Tool for the Automatic Analysis of Cohesion (TAACO) 2.0.4

ID	Picture	Transcribe speech	Repeated contents lemmas (Givenness)	Repeated_content_and_pronoun_lemmas (Givenness)
FEPxxx	1	Um he is looking at an enemy who is done wrong to him and she is trying to console him they are both of uh decent socioeconomic status they have nice clothing nicely cropped hair and uh he is probably he is probably under the influence of alcohol and uh I think he like there is something going on underneath the surface for him that she does not know about but she is still there trying to, trying to face things for him there is a woman in the background so that probably suggests that um I do not know	0.160	0.320

Note. MLS: mean length of sentences; MLT: mean length of T-units; MLC: mean length of clauses. Givenness: It is an average number of content words that are repeated at least once divided by the total number of words in the text.

3 Hierarchical Clustering with Patients Only

When we clustered both patients and controls, there were naturally two categories of participants, and therefore a 2-cluster solution may be simply detecting a dominant effect. To rule out this possibility and confirm that a 2-cluster solution remains in the patient sample only, we ran the same clustering procedure for 66 patients. The results showed that a two-cluster solution still received the highest number of votes.

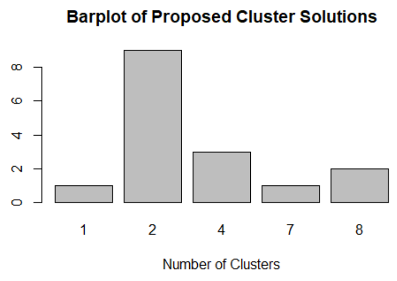

4 Comparisons of patients from the two subgroups

4.1 Tables

Supplementary Table 1. Linguistic data patient subgroup comparisons when considering age effects in a linear covariance model

	Subgroup 1 Patients	Subgroup 2 Patients	
**N**	46	20	
**Language Variables**			**ANOVA with age as a covariate**
TLI (Total)	1.28 (1.28)	1.93 (1.64)	F(1)=2.96, p = 0.090<br>Age effect: p = 0.39
TLI Impoverishment	0.48 (0.61)	0.79 (0.92)	F(1)=2.61, p = 0.11<br>Age effect: p = 0.29
TLI Disorganization	0.82 (1.14)	1.14 (1.37)	F(1)=1.00, p = 0.32<br>Age effect: p = 0.15
Average total number of words	119.47 (35.45)	118.43 (47.46)	F(1)=0.009, p = 0.92<br>Age effect: p = 0.126
MLS	14.58 (4.01)	13.91 (5.89)	F(1)=0.25, p = 0.62<br>Age effect: p = 0.25
MLT	12.79 (3.09)	10.75 (2.20)	F(1)=6.46, p = 0.014 *<br>Age effect: p = 0.57
MLC	7.90 (1.25)	7.30 (0.96)	F(1)=3.30, p = 0.074<br>Age effect: p = 0.126
Repeated contents lemmas	0.240 (0.044)	0.204 (0.047)	F(1)=7.56, p = 0.0081 **<br>Age effect: p = 0.515

Note: Values are reported as Mean (SD). TLI: Thought and Language Index; MLS: mean length of sentences, MLT: mean length of T-units, MLC: mean length of clauses.

* p values < 0.05

** p values < 0.01

*** p values < 0.001

Supplementary Table 2. Cortical regions with their area size (mm^2) showed significant differences after Monte Carlo simulation correction between patients from the two subgroups, in the left and right hemispheres respectively.

	Left Hemisphere	Right Hemisphere
**Inferior temporal**	1) 1082.27	
**Lateral orbitofrontal**	2) 579.75<br>3) 530.65	
**Rostral middle frontal**	4) 462.95	1) 560.93<br>2) 260.59
**Precentral**	5) 389.09	3) 420.84<br>4) 247.97
**Precuneus**	6) 289.00	
**Rostral anterior cingulate**	7) 234.62	
**Postcentral**	8) 182.27	
**Lateral occipital**		5) 437.17<br>6) 333.73<br>7) 266.67
**Lingual**		8) 258.06

4.2 Figures

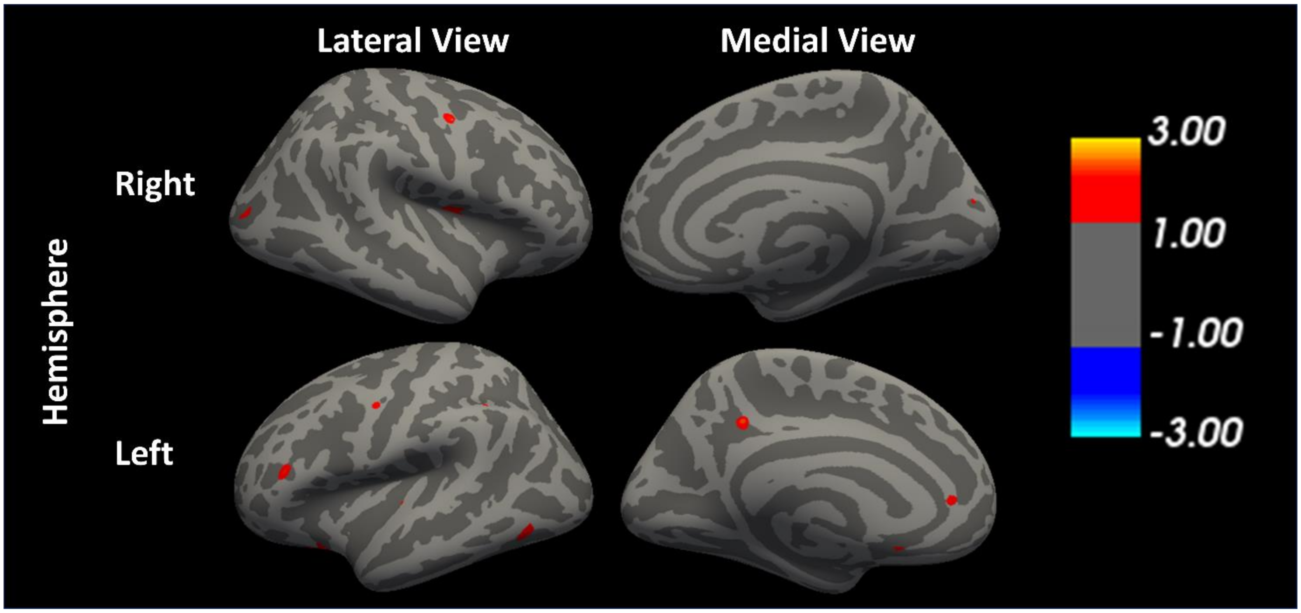

Supplementary Figures 1. Cortical thickness map of differences between patients from Subgroup 1 and Subgroup 2 generated by FreeSurfer (regressing out age effect with a general linear model, multiple comparison corrections using Monte Carlo simulations of 1000 permutations with a cluster-wise threshold of 0.05). Left hemisphere and right hemisphere in lateral and medial view

respectively. The scale indicates $\log_{10}$ of p-values. Red and yellow represent higher cortical thickness in patients from Subgroup 1.

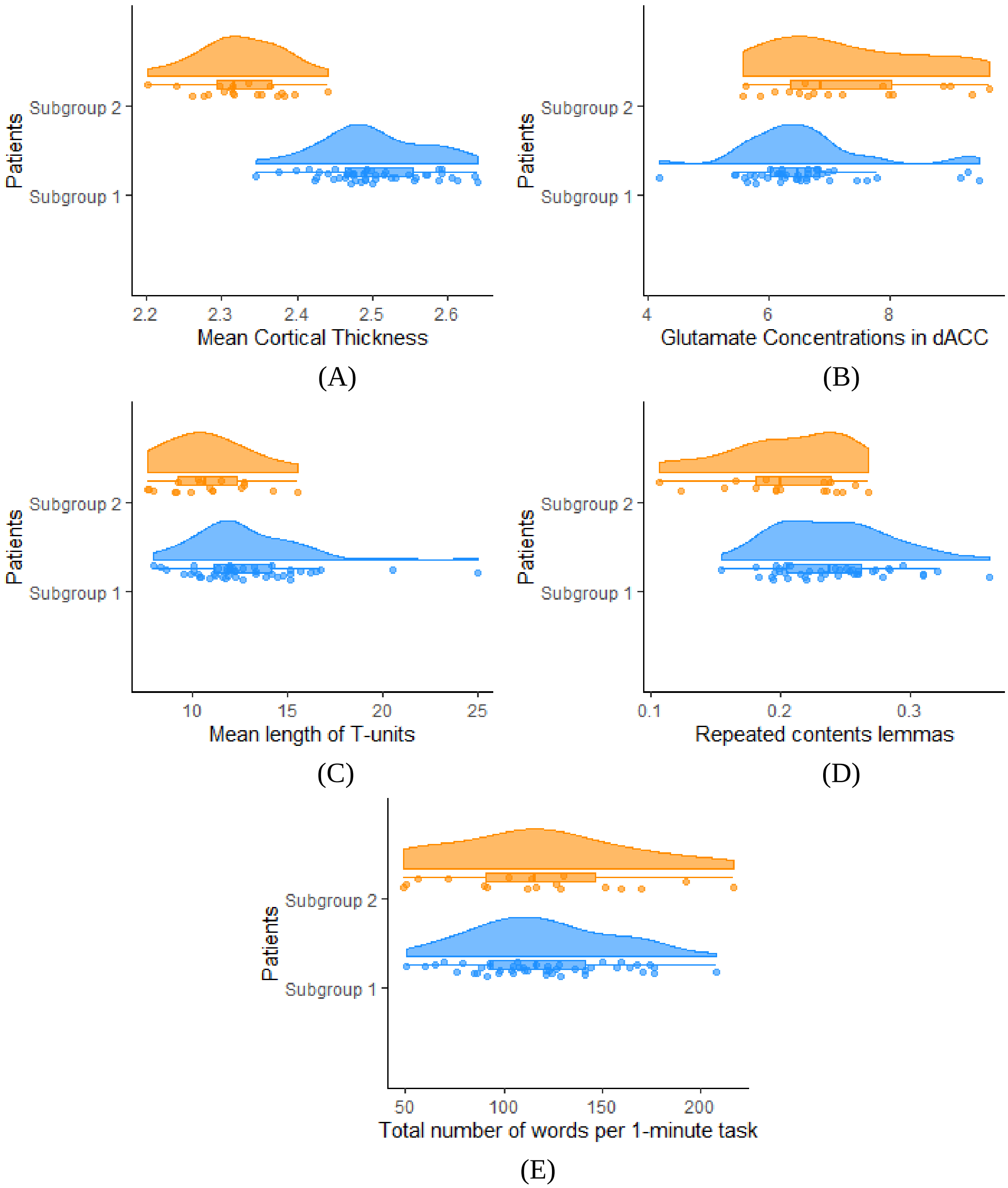

Supplementary Figures 2. Raincloud plots depicting the comparisons of distributions between the two patient subgroups.

5 Correlation Matrices

We used number of words to index fluency; MLS/MLT/MLC to index syntactic complexity; and repeated content lemmas (RCL) to index cohesion of patients' speech discourse. For the whole patient sample, the correlation coefficients showed that total number of words was weakly correlated with the other two measurements (positive correlation with MLT, Pearson's correlation R = 0.28, p = 0.028; positive correlation with RCL, Pearson's correlation R = 0.33, p = 0.0068; Supplementary Figure 3B). However, syntactic complexity metrics and cohesions metric were independent of one another. The clinical rating scale of language disorder (TLI) was not significantly correlated with any of the language measurements we selected (Supplementary Figure 3B).

Although glutamate was not correlated with the language indices for subgroup 1 patients (Supplementary Figure 3C), glutamate was negatively correlated particularly with MLC (R=-0.45) for the subgroup of patients with widespread cortical thinning, which further supports the role of glutamate in language dysfunctions in a subgroup of schizophrenia (1). A negative correlation between PANSS negative symptom severity and glutamate levels was also only restricted to the 'cortical thinning' subgroup, in line with other studies that discussed associations between glutamate and negative symptom severity (2).

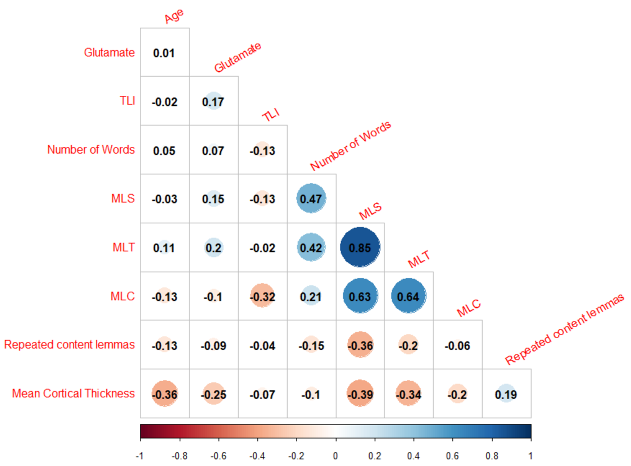

(A)

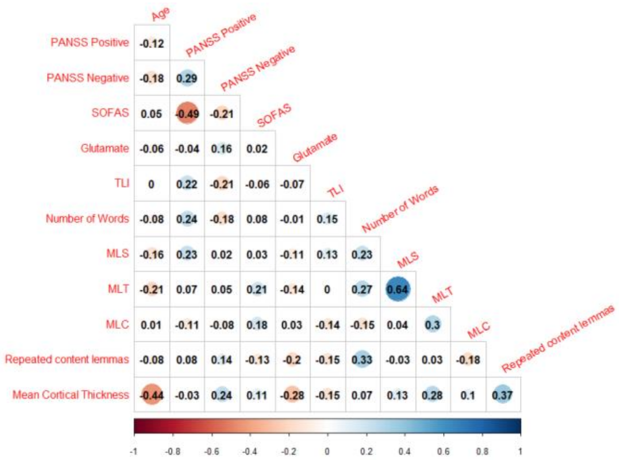

(B)

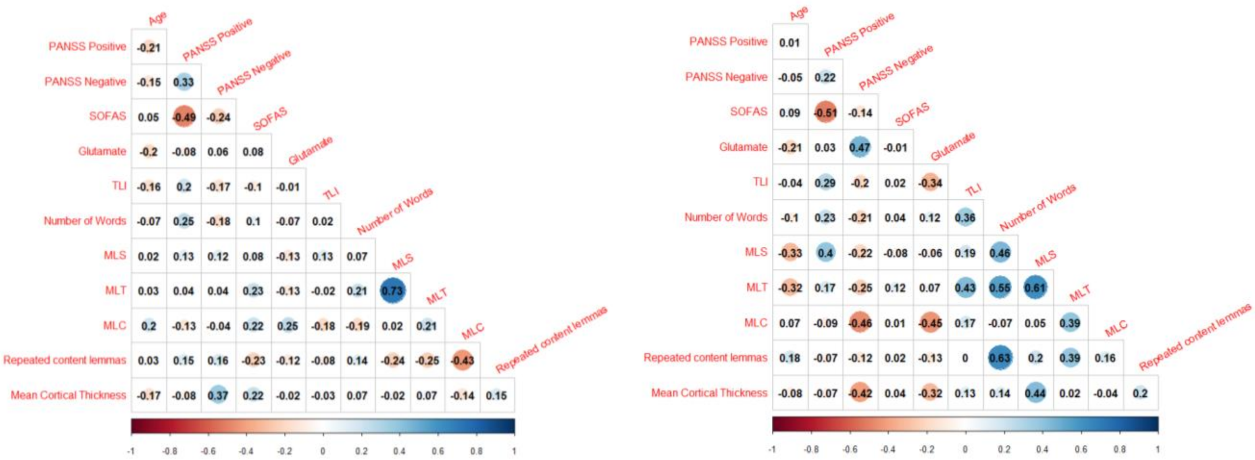

(C) (D)

Supplementary Figures 3. Correlations between variables of interest of (A) healthy controls; (B) all patients; (C) Subgroup 1 patients with near-normal cortical thickness (n=46); (D) Subgroup 2 patients with widespread cortical thinning (n = 20), respectively. The numbers indicate correlation coefficients between two variables. The circle size indicates strengths of correlations while the colours indicate directions of correlations (blue: positive correlation; red: negative correlation).

6 MRS Acquisition and Processing

A long echo-time semi-LASER 1H-MRS pulse sequence (TR=7500ms, TE=100ms) was used to acquire 32 channel-combined, water-suppressed spectra. Each spectra underwent phase and frequency correction (3) before being averaged into a single representative spectrum. We then performed QUECC (4), HSVD (5) water removal, and spectral fitting using fitMAN (6), a time-domain fitting algorithm that uses a nonlinear, iterative Levenberg-Marquardt minimization algorithm to estimate the chemical shift, amplitude, linewidth and phase of echo-time specific prior knowledge templates. Our fitting template consisted of 17 brain metabolites (described in more detail in our previous work (7)). The fitted spectrum was then used in Barstool (8) to correct for CSF and gray and white matter volumes and finally for calculating metabolite concentration estimates. See tissue fractions in Supplementary Table 3. All spectra were visually inspected for quality assurance. Additionally, only those metabolites with Cramer-Rao lower bounds (CRLB) lower than 10% were included in the analysis. SNR, defined as the ratio between signal amplitude of the NAA peak in the frequency domain divided and the standard deviation of the noise in the last 32 most up-field points of the spectrum, was calculated to be 108.0 ± 20.8 for all participants. SNR were calculated as follows: Healthy Control 112.39 ± 15.15 VS. FEP 105.70 ± 22.99 [t(88) = 1.653, p = 0.102].

Supplementary Table 4. Tissue volume fractions in the voxel placed in dorsal anterior cingulate cortex.

	First-episode psychosis (N = 66)	Healthy controls (N = 36)	Total (N = 102)
Grey matter	0.5590 ± 0.0620	0.5911 ± 0.0502	0.5701 ± 0.0599
White matter	0.2041 ± 0.0706	0.1961 ± 0.0379	0.2014 ± 0.0612

Cerebrospinal fluid	0.2367 ± 0.0740	0.2128 ± 0.0591	0.2285 ± 0.0698

Note: Values are reported as mean ± standard deviation of tissue proportion in the voxel.

Supplementary Table 5. Description of single voxel ^{1}H-MRS of MRS hardware, data acquisition, analysis, and quality assessment details.

1. Hardware	
a. Field strength [T]	7-Tesla
b. Manufacturer	Siemens
c. Model (software version if available)	VB17
d. RF coils: nuclei (transmit/receive), number of channels, type, body part	32 channel head coil (8-channel Tx, 32-channel Rx)
e. Additional hardware	N/A
**2. Acquisition**	
a. Pulse sequence	semi-LASER
b. Volume of Interest (VOI) locations	Bilateral dorsal anterior cingulate cortex
c. Nominal VOI size [cm^3, mm^3]	2 x 2 x 2 cm^3
d. Repetition Time (TR), Echo Time (TE) [ms,s]	TR = 7500ms, TE = 100ms
e. Total number of excitations or acquisitions per spectrum	32 averages, 1 measurement
f. Additional sequence parameters (spectral width in Hz, number of spectral points, frequency offsets)	2048 points
g. Water Suppression Method	VAPOR
h. Shimming Method, reference peak, and thresholds for “acceptance of shim” chosen	FASTESTMAP
i. Triggering or motion correction method	N/A
**3. Data analysis methods and outputs**	
a. Analysis software	MATLAB, fitMAN, Barstool
b. Processing steps deviating from quoted reference or product	N/A
c. Output measure (e.g. absolute concentration, institutional units, ratio)	Absolute concentration
d. Quantification references and assumptions, fitting model assumptions	Each spectrum was phase and frequency corrected to the first spectral acquisition before being averaged into a single spectrum for further post-processing. 17 brain metabolites (described in Methods) were included our fitting template and quantification analysis.
**4. Data Quality**	

a. Reported variables<br>(SNR, Linewidth (with reference peaks))	SNR
b. Data exclusion criteria	No subjects excluded
c. Quality measures of postprocessing Model fitting<br>(e.g. CRLB, goodness of fit, SD of residual)	CRLB
d. Sample Spectrum	See Supplementary Figure 4

Note: This table was based on a MRS reporting standardized template provided by Lin et al. (9)

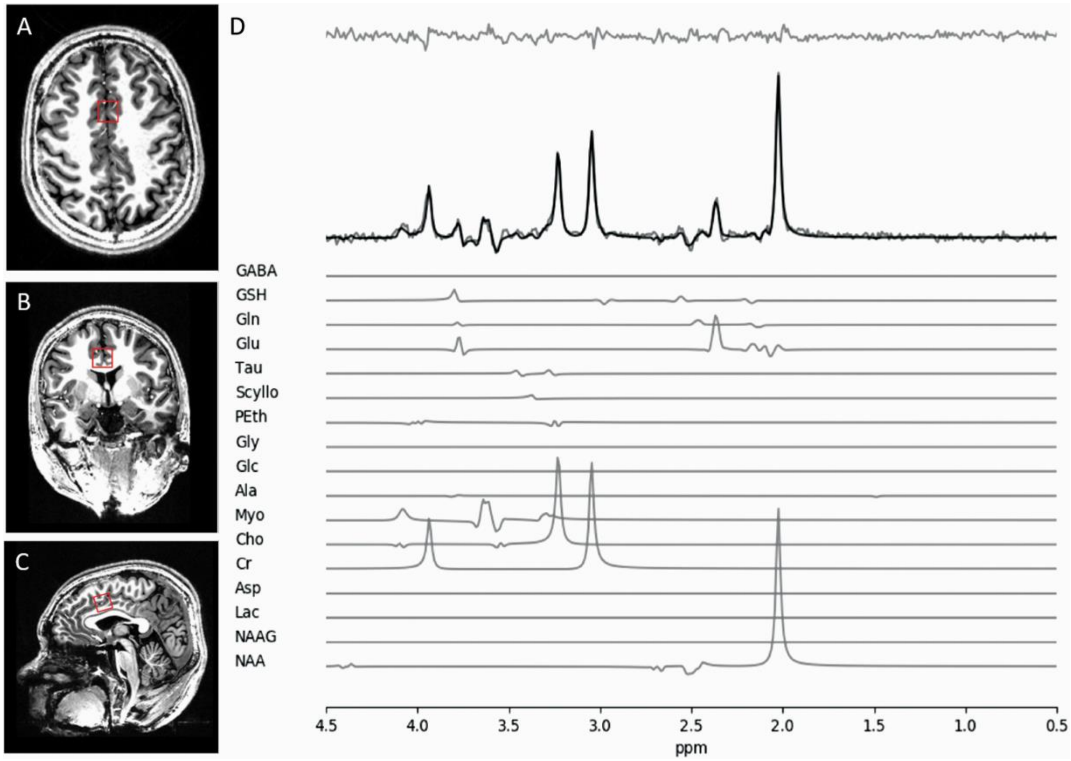

Supplementary Figure 4. MRS voxel location and an exemplar spectrum. (A) axial, (B) coronal, and (C) sagittal views of MRS voxel (red square) in the dorsolateral anterior cingulate cortex (ACC) for glutamate measurement. (D) Sample fitted spectrum of a single participant. Fit spectrum (bolded) is overlaid on the raw spectrum with the residual spectrum displayed above. Individual component spectra of all 17 template-included metabolites are displayed below.
Note: This figure was previously published in Schizophrenia Bulletin (https://academic.oup.com/schizbullopen/article/2/1/sgaa072/6126062?login=true) and was originally created by one of the co-authors PJ. This image was included here with the permission of PJ.

7 Bash Scripts and R Codes

Bash script to reconstruct brain surfaces and calculate vertex-wise thickness values in FreeSurfer

```
#!/usr/bin/env bash
export SUBJECTS_DIR=/media/sf_subjects/recon

for subj in `ls ./nifti`
do
    recon-all -s $subj -i ./nifti/$subj/*.nii -all -qcache
done
```

Bash script to extract thickness values based on Destrieux parcellation (10)

```
# define subjects data directory path
export SUBJECTS_DIR=/home/charlotte/Desktop/recon

# output stats from recon-all
aparcstats2table --hemi lh \
    --meas thickness \
    --parc aparc.a2009s \
    --tablefile 211108_lh_thicknes_destrieux.txt \
    --subjects

aparcstats2table --hemi rh \
    --meas thickness \
    --parc aparc.a2009s \
    --tablefile 211108_rh_thicknes_destrieux.txt \
    --subjects
```

Bash script to output cortical thickness map of differences between two subgroups (regressing out age effect with a general linear model, multiple comparison corrections using Monte Carlo

```
export SUBJECTS_DIR=/home/charlotte/Desktop/recon
cat group_diff.fsgd | sed 's/\r/\n/g' > new.group_diff.fsgd

# Resampling subjects data into a common space; spatial soothing
mris_preproc --fsgd new.group_diff.fsgd --target fsaverage --hemi lh --meas thickness --out
lh_group_diff.mgh
mris_preproc --fsgd new.group_diff.fsgd --target fsaverage --hemi rh --meas thickness --out
rh_group_diff.mgh

# GLM model fit
mri_glmfit --y lh_group_diff.mgh --fsgd new.group_diff.fsgd --C group_diff.mtx --glmdir
group.age_10sm.lh --fwhm 10 --surface fsaverage lh --eres-save
```

simulations of 1000 permutations with a cluster-wise threshold of 0.05)	<pre>mri_glmfit --y rh_group_diff.mgh --fsgd new.group_diff.fsgd --C group_diff.mtx --glmdir group.age_10sm.rh --fwhm 10 --surface fsaverage rh --eres-save<br><br># Multiple testing correction<br>mri_glmfit-sim --glmdir group.age_10sm.lh --2spaces --cwp 0.05 --perm 1000 3 abs<br>mri_glmfit-sim --glmdir group.age_10sm.rh --2spaces --cwp 0.05 --perm 1000 3 abs</pre>
R codes to run clustering procedure and other statistical analyses	<pre>#Import dataset generated by FreeSurfer----<br>TOPSY <- read_excel("E:/subjects/Bash Scripts/TOPSY_destrieux_thickness_211108_66FEP36HC.xlsx")<br>TOPSY_thickness <- as.data.frame(TOPSY[c(37:184)])<br>rownames(TOPSY_thickness) <- TOPSY$ID<br><br># Use original thickness values for clustering ----<br># Hierarchical Cluster Analysis<br>TOPSY_dist <- dist(TOPSY_thickness, method = "euclidean")<br>TOPSY_hc_ward <- hclust(TOPSY_dist, method = "ward.D2")<br>TOPSY_cluster_solution <- matrix(rep(0, len=length(selected)),nrow = length(selected))<br>for (i in 1:length(selected)){<br>TOPSY_cluster_solution[i,] <- unname(NbClust::NbClust(TOPSY_thickness, min.nc=1, max.nc=8, method="ward.D2", index=selected[i])$Best.nc)[1]<br>}<br>barplot(table(TOPSY_cluster_solution), main = "Barplot of Proposed Cluster Solutions",xlab="Number of Clusters")<br>plot(TOPSY_hc_ward)<br>rect.hclust(TOPSY_hc_ward, k = 2)<br>TOPSY_2clusters <- cutree(TOPSY_hc_ward, k=2)<br><br># Subgroups Statistics<br>TOPSY$cluster = TOPSY_2clusters<br><br># Explore two-cluster solution<br>TOPSY_TypeCluster <- table(data.frame(TOPSY$Type,TOPSY$cluster))<br>barplot(TOPSY_TypeCluster,xlab="cluster assignment", ylab="patient or control",<br>main="Patient & control in each cluster",legend=rownames(TOPSY_TypeCluster))<br>chisq.test(TOPSY_TypeCluster)<br>write.csv(TOPSY_FEP,"E:/TOPSY/TOPSY_FEP_FULL.csv")<br><br># Use original thickness and clustering with FEP only ----<br># Hierarchical Cluster Analysis<br>TOPSY_FEP_thickness <- as.data.frame(TOPSY_FEP[c(37:184)])<br>TOPSY_dist <- dist(TOPSY_FEP_thickness, method = "euclidean")</pre>

	```<br>TOPSY_hc_ward <- hclust(TOPSY_dist, method = "ward.D2")<br>TOPSY_cluster_solution <- matrix(rep(0, len=length(selected)),nrow = length(selected))<br>for (i in 1:length(selected)){<br>TOPSY_cluster_solution[i,] <- unname(NbClust::NbClust(TOPSY_thickness, min.nc=1, max.nc=8, method="ward.D2", index=selected[i])$Best.nc)[1]<br>}<br>barplot(table(TOPSY_cluster_solution), main = "Barplot of Proposed Cluster Solutions",xlab="Number of Clusters")<br>plot(TOPSY_hc_ward)<br>rect.hclust(TOPSY_hc_ward, k = 2)<br>TOPSY_2clusters <- cutree(TOPSY_hc_ward, k=2)<br><br># Subgroups Statistics<br>TOPSY_FEP$cluster_FEP = TOPSY_2clusters<br><br># Check cluster consistency<br>cluster_consistency_table <- table(data.frame(TOPSY_FEP$cluster,TOPSY_FEP$cluster_FEP))<br>barplot(cluster_consistency_table, xlab="x", ylab="y", main="Cluster Consistency")<br><br># Correlations between symptom and language scores ----<br>TOPSY_Language2$MeanThickness <- TOPSY$meanThickness<br>CorMatrix <- TOPSY_Language2[,c(3,7:8,18,24,30,40:43,60,62)] #variables of all participants<br><br>corrplot.mixed(cor(CorMatrix, method = "pearson", use = "pairwise.complete.obs"))<br>corrplot(cor(CorMatrix, method = "pearson", use = "pairwise.complete.obs"),addCoef.col = 'black',type = 'lower',diag = FALSE)<br><br>CorMatrix_FEP <- subset(TOPSY_Language2, Type =="FEP")[,c(3,7:8,18,24,30,40:43,60,62)] #variables of FEP only<br>colnames(CorMatrix_FEP) = c("Age", "PANSS Positive", "PANSS Negative","SOFAS","Glutamate","TLI","Number of Words",<br>"MLS","MLT","MLC","Repeated content lemmas", "Mean Cortical Thickness")<br>corrplot(cor(CorMatrix_FEP, method = "pearson", use = "pairwise.complete.obs"),addCoef.col = 'black',type = 'lower',diag = FALSE,tl.srt = 30)<br><br>CorMatrix_FEP1 <- subset(subset(TOPSY_Language2, Type =="FEP"),cluster==1)[c(3,7:8,18,24,30,40:43,60,62)] #variables of FEP1 only<br>colnames(CorMatrix_FEP1) = c("Age", "PANSS Positive", "PANSS Negative","SOFAS","Glutamate","TLI","Number of Words",<br>```

	```<br>"MLS","MLT","MLC","Repeated content lemmas", "Mean Cortical Thickness")<br>corrplot(cor(CorMatrix_FEP1, method = "pearson", use = "pairwise.complete.obs"),addCoef.col = 'black',type = 'lower',diag = FALSE,tl.srt = 30)<br><br>CorMatrix_FEP2 <- subset(subset(TOPSY_Language2, Type =="FEP"),cluster==2)[c(3,7:8,18,24,30,40:43,60,62)] #variables of FEP2 only<br>colnames(CorMatrix_FEP2) = c("Age", "PANSS Positive", "PANSS Negative","SOFAS","Glutamate","TLI","Number of Words",<br>"MLS","MLT","MLC","Repeated content lemmas", "Mean Cortical Thickness")<br>corrplot(cor(CorMatrix_FEP2, method = "pearson", use = "pairwise.complete.obs"),addCoef.col = 'black',type = 'lower',diag = FALSE,tl.srt = 30)<br><br>CorMatrix_HC <- subset(TOPSY_Language2, Type =="HC")[c(3,24,30,40:43,60,62)] #variables of HC only<br>colnames(CorMatrix_HC) = c("Age", "Glutamate","TLI","Number of Words",<br>"MLS","MLT","MLC","Repeated content lemmas", "Mean Cortical Thickness")<br>corrplot(cor(CorMatrix_HC, method = "pearson", use = "pairwise.complete.obs"),addCoef.col = 'black',type = 'lower',diag = FALSE,tl.srt = 30)<br><br># Raincloud plots for variables ----<br>remotes::install_github('jorvlan/raincloudplots')<br>library(raincloudplots)<br><br>#Define plotting raincloud plot function<br>Plot_raincloud <- function(variable){<br>variable_rain <- data_1x1(array_1 = subset(subset(TOPSY_Language2, cluster==1),Type=="FEP")[[variable]],<br>array_2 = subset(subset(TOPSY_Language2, cluster==2),Type=="FEP")[[variable]],<br>jit_distance = 0.2,<br>jit_seed = 321)<br>variable_raincloud <- raincloud_1x1(data=variable_rain,<br>#colors = (c('dodgerblue','darkorange')),<br>#fills = (c('dodgerblue','darkorange')),<br>size = 1.5,<br>alpha = .6,<br>ort = 'h') +<br>scale_x_continuous(breaks=c(1,2), labels=c("Subgroup 1", "Subgroup 2"), limits=c(0, 3)) +<br>xlab("Patients") +<br>```

	<pre> theme_classic()<br>return(variable_raincloud)<br>}<br><br>#Glutamate raincloud<br>Glu_raincloud <- Plot_raincloud(variable = "Rest_Glu")<br>Glu_raincloud + ylab("Glutamate Concentrations in dACC")<br>#Thickness raincloud<br>Thickness_raincloud <- Plot_raincloud(variable = "MeanThickness")<br>Thickness_raincloud + ylab("Mean Cortical Thickness")<br>#Age raincloud<br>Age_raincloud <- Plot_raincloud(variable = "Age")<br>Age_raincloud + ylab("Age")<br>#MLT raincloud<br>MLT_raincloud <- Plot_raincloud(variable = "Mean-MLT")<br>MLT_raincloud + ylab("Mean length of T-units")<br>#repeated contents lemmas raincloud<br>RCL_raincloud <- Plot_raincloud(variable = "repeated_content_lemmas")<br>RCL_raincloud + ylab("Repeated contents lemmas")<br>#total words raincloud<br>words_raincloud <- Plot_raincloud(variable = "Mean-nwords")<br>words_raincloud + ylab("Total number of words per 1-minute task")<br>#DUP&DDD distribution<br>ggplot(TOPSY_Language2_patient, aes(x=DUP_Weeks, fill = cluster)) + geom_density(alpha=.3) +<br>xlim(0,120) + xlab("Duration of untreated psychosis in weeks")<br>ggplot(TOPSY_Language2_patient, aes(x=DDD_LifeTime, colour = cluster)) + geom_density() +<br>xlim(0,25) + xlab("DDD lifetime exposure")</pre>

8 Reference

1. Palaniyappan L. Dissecting the neurobiology of linguistic disorganisation and impoverishment in schizophrenia. Semin Cell Dev Biol [Internet]. 2021 Sep 8 [cited 2022 Feb 1]; Available from: https://www.sciencedirect.com/science/article/pii/S1084952121002354

2. Gruber O, Santuccione Chadha A, Aach H. Magnetic Resonance Imaging in Studying Schizophrenia, Negative Symptoms, and the Glutamate System. Front Psychiatry [Internet]. 2014 [cited 2022 May 24];5. Available from: https://www.frontiersin.org/article/10.3389/fpsyt.2014.00032

3. Near J, Edden R, Evans CJ, Paquin R, Harris A, Jezzard P. Frequency and phase drift correction of magnetic resonance spectroscopy data by spectral registration in the time domain. Magn Reson Med. 2015 Jan;73(1):44–50.

4. Bartha R, Drost DJ, Menon RS, Williamson PC. Spectroscopic lineshape correction by QUECC: combined QUALITY deconvolution and eddy current correction. Magn Reson Med. 2000 Oct;44(4):641–5.

5. van den Boogaart A, Ala-Korpela M, Jokisaari J, Griffiths JR. Time and frequency domain analysis of NMR data compared: an application to 1D 1H spectra of lipoproteins. Magn Reson Med. 1994 Apr;31(4):347–58.

6. Bartha R, Drost DJ, Williamson PC. Factors affecting the quantification of short echo in-vivo 1H MR spectra: prior knowledge, peak elimination, and filtering. NMR Biomed. 1999 Jun;12(4):205–16.

7. Jeon P, Limongi R, Ford SD, Mackinley M, Dempster K, Théberge J, et al. Progressive Changes in Glutamate Concentration in Early Stages of Schizophrenia: A Longitudinal 7-Tesla MRS Study. Schizophr Bull Open. 2021 Jan 1;2(1):sgaa072.

8. Wong D. MRI Investigations of Metabolic and Structural Brain Changes in Alzheimer’s Disease and Vitamin D Deprivation. Electron Thesis Diss Repos [Internet]. 2019 Sep 6; Available from: https://ir.lib.uwo.ca/etd/6611

9. Lin A, Andronesi O, Bogner W, Choi I, Coello E, Cudalbu C, et al. Minimum Reporting Standards for in vivo Magnetic Resonance Spectroscopy (MRSinMRS): Experts’ consensus recommendations. Nmr Biomed. 2021 May;34(5):e4484.

10. Destrieux C, Fischl B, Dale A, Halgren E. Automatic parcellation of human cortical gyri and sulci using standard anatomical nomenclature. NeuroImage. 2010 Oct 15;53(1):1–15.
